# Neurochemical Measurement of Adenosine in Discrete Brain Regions of Five Strains of Inbred Mice

**DOI:** 10.1371/journal.pone.0092422

**Published:** 2014-03-18

**Authors:** Amar K. Pani, Yun Jiao, Kenneth J. Sample, Richard J. Smeyne

**Affiliations:** Department of Developmental Neurobiology, St Jude Children’s Research Hospital, Memphis, Tennessee, United States of America; Emory University, United States of America

## Abstract

Adenosine (ADO), a non-classical neurotransmitter and neuromodulator, and its metabolites adenosine triphosphate (ATP), adenosine diphosphate (ADP) and adenosine monophosphate (AMP), have been shown to play an important role in a number of biochemical processes. Although their signaling is well described, it has been difficult to directly, accurately and simultaneously quantitate these purines in tissue or fluids. Here, we describe a novel method for measuring adenosine (ADO) and its metabolites using high performance liquid chromatography with electrochemical detection (HPLC-ECD). Using this chromatographic technique, we examined baseline levels of ADO and ATP, ADP and AMP in 6 different brain regions of the C57BL/6J mouse: stratum, cortex, hippocampus, olfactory bulb, substantia nigra and cerebellum and compared ADO levels in 5 different strains of mice (C57BL/6J, Swiss-Webster, FVB/NJ, 129P/J, and BALB/c). These studies demonstrate that baseline levels of purines vary significantly among the brain regions as well as between different mouse strains. These dissimilarities in purine concentrations may explain the variable phenotypes among background strains described in neurological disease models.

## Introduction

Purine and pyrimidine nucleosides and bases, the essential building blocks of nucleic acids, occur widely throughout the animal kingdom and underlie a number of critical functions including energy transduction, metabolism and cell signaling. One endogenous purine nucleoside, adenosine (ADO), plays an important role in a number of biochemical processes including energy transfer—as adenosine triphosphate (ATP) and adenosine diphosphate (ADP) as well as signal transduction; contained within cyclic adenosine monophosphate (cAMP).

In the nervous system, ADO acts as a non-classical inhibitory neurotransmitter [1], [2] and neuromodulator [3], [4]. Alterations in ADO or its signaling have been linked to a number of neurological disorders including epilepsy [5], Parkinson’s disease [6], [7], schizophrenia [8], panic disorder and anxiety [9], as well as drug abuse [10]. Alterations in ADO have also been linked to changes in a sleep and arousal [11] as well as cognition and memory [12], [13].

Within the brain, extracellular ADO concentrations have been reported to be in the 30–400 nM range [14]–[16]. However, in response to cellular damage (e.g. seizure or ischemia), these concentrations can quickly elevate [16]–[18], in some cases 7.5-31-fold [19], suggesting that ADO, in addition to signaling, also can have a neuroprotective function.

Adenosine functions by binding to, and signaling through, four known receptor subtypes (A1, A2A, A2B, and A3) [20], [21]. One of the best known compounds that acts via ADO signaling, and in particular by binding to the A2A receptor, is caffeine [22]. This drug’s stimulatory effects are primarily (although not entirely) credited to its inhibition of ADO via competitive inhibition of these receptors [23], effectively blocking ADO signaling. The subsequent reduction in ADO signaling leads to increased activity of other neurotransmitters including acetylcholine [24] noradrenaline [25], GABA [26], dopamine [27], and glutamate [28].

Although our understanding of the synaptic mechanisms that underlie ADO neurotransmission in mammals has greatly advanced, progress in simultaneously separating and identifying individual purines and demonstration of their specific neurochemical functions have been unsatisfactory. This paucity in information is due, in part, to the lack of a standardized analytical method to directly and accurately measure total tissue levels of ADO and its metabolites. To this end, we have developed a new quantitative protocol that takes advantage of high performance liquid chromatography with electrochemical detection (HPLC-ECD) to directly measure ADO and its metabolites ATP, ADP and AMP.

Given that ADO is implicated in a number of neurological disorders, we quantified baseline levels of ADO, ATP, ADP and AMP from six different CNS regions and compared the levels of ADO in these different brain regions in five different strains of mice commonly used in the generation or study of neurologic disease models. We find that ADO levels significantly vary dependent on both the regions and mouse strain examined. These differences appear to correlate with functional differences between strains and may provide information that will be useful in the generating animal models of neurodegenerative disorders.

## Methods

### Ethics Statement

All animals used in this study were treated in accordance with NIH guidelines and approved by the St Jude Children’s Research ACUC under IACUC protocol number 364.

### Animals

Male C57BL/6J, FVB/NJ, 129P/J and BALB/c mice were purchased from the Jackson Labs (Bar Harbor, ME), while Swiss Webster (SW) mice were purchased from Harlan (Indianapolis, IN). Mice were housed five per cage in the AALAC certified vivarium at St Jude Children’s Research Hospital. Mice were maintained on a 12 hour light-dark cycle (6:00 AM-6:00 PM) in a temperature and humidity controlled room with food and water ad libitum. All of the mice in this study were 6–12 months old and weighed between 23–29 g at the time of sacrifice.

### Chemicals

Optima LC/MS grade acetonitrile, methanol, perchloric acid and phosphoric acid were purchased from Thermo-Fisher Scientific (NJ). Adenosine (ADO), adenosine 5′-diphosphate monopotassium salt (ADP), adenosine 5′-monophosphate (AMP), sodium dihydrogen phosphate, potassium phosphate, sodium acetate and sodium perchlorate were purchased from Sigma-Aldrich Chemical Co. (St. Louis, MO). Adenosine triphosphate disodium (ATP, Cat # A1319) was purchased from LKT laboratories, St. Paul, MN).

### Preparation of Tissue for HPLC-ECD analysis of Adenosine

Mice were deeply anesthetized with Avertin until all deep tendon and corneal reflexes were absent and immediately transcardially perfused with ice-cold saline to remove circulating blood. Following perfusion, brains were rapidly dissected from the calvaria, placed in a cooled mouse brain matrix (Model BS-AL-5000C, Braintree Scientific, Braintree, MA) and sliced into 2 mm thick sections. Six brain regions were rapidly subdissected using the following coordinates [29]: olfactory bulb, cerebral cortex (Bregma: –1.00– –3.00 mm), striatum (Bregma: +0.00–+2.00 mm), hippocampus (Bregma: –1.00– –3.00 mm), substantia nigra (Bregma: –2.00– –4.00) and brainstem (Bregma: –5.00– –7.00). This whole process took approximately 7 minutes/mouse.

Once the tissues were dissected, the samples were immediately frozen on dry ice, placed into pre-weighed 1.5 ml Eppendorf tubes and stored at –80°C. On the day of analysis, tissue was weighed, then thawed and minced in 200 microliters of Pani mobile phase, (see below). Tissue samples were homogenized (Pellet Pestle Motor, Thermo- Fisher Scientific) and centrifuged at 13,700 rpm for 27 minutes at 4°C. The homogenized samples were vacuum filtered using a 96 well Millipore Vacuum Manifold Unit with DirectStack Technology (Millipore, Cat# MSVMHTS00). The resultant pellet and supernatant were separated, then frozen and stored at –80°C for no more than two weeks prior to the chromatographic analysis.

### Preparation of Standard Solutions and Biological Sample

Separate stock standard solutions of ADO, ADP and AMP were prepared in Pani mobile phase at 1 mg/ml, while ATP was prepared at 10 mg/ml. Each were stored at 4°C. The working standard solutions were prepared daily by diluting the stock solutions to serial dilutions of 10 mg/ml. 5/mg/ml, 2.5 mg/ml and 1.25 mg/ml. 10 ul of a mixed standard, containing equal amounts of ADO, ATP, ADP and AMP, solution was injected using an autosampler to generate a simultaneous standard curve immediately prior to HPLC-ECD analysis of the brain samples.

The brain samples were prepared using a standard diluent comprised of Pani mobile phase (75%) with 0.2N perchloric acid (25%), which gave a recovery of approximately 90% compared to other standard diluents (Figure S1).

### Comparison of ADO separation and quantification using different columns and mobile phases

Two of the key determinant factors that influence separation and quantification of purines in HPLC-ECD is use of the stationary phase (chromatographic column) in combination with the proper electrolyte (mobile phase). Selection of the proper mobile phase is critical since one has to properly dissolve the compounds to be measured, while the proper column functions to differentially retain solutes based upon hydrophobic interactions with its stationary phase.

During development of our assay, we discovered 2 different published protocols describing HPLC-ECD identification of adenosine [30], [31]. However, neither of these papers described the direct and simultaneous detection of adenosine as well as its metabolites, ATP, ADP and AMP. Thus, we began development of a novel protocol. During the development of this HPLC-ECD protocol for simultaneous detection of ADO, ATP, ADP and AMP we empirically tested 4 different columns of various lengths and 5 different mobile phases based either on the parameters described in these previous papers or the known electrochemical properties of purines (oxidative potential, column retention and chromatographic separation) of these combinations. Each column and mobile phase was tried in combination, so that 20 possible pairs were tested.

The columns tested were: 1) ESA, HR80, 4.6×80 mm, C18, 3 micron particle size (part# 68-0100); ESA, MD-150, 3.2×150 mm, C18, 3 micron particle size (part# 70-0636); Kinetex 2.1×50 mm, C18, 2.6 micron particle size (part# 00B-4496-AN), and ESA, DHBA-3.2×250 mm, C18, 5 micron particle size (part# 70-2115).

The mobile phases tested were: 1) Sodium phosphate monobasic & dibasic, 1-Octanesulphonic acid, acetonitrile and methanol, pH 3.85; 2) Sodium phosphate monobasic, 1-Octanesulphonic acid; acetonitrile, pH 3.85, 3) Potassium phosphate, 1-Octanesulphonic acid, EDTA, acetonitrile and methanol, pH 3.5; 4) Sodium phosphate, Potassium phosphate, 1-Octanesulphonic acid, EDTA, methanol and acetonitrile, pH 3.5; 5) A novel mobile phase (Pani mobile phase) that consisted of 19.0 mM sodium perchlorate (Fischer Scientific) and 4.0% (v/v) Acetonitrile (Optima, LC/MS grade) prepared in double distilled, deionizer, autoclaved water tested at 4 different pH values (1.75, 2.75, 3.75 and 7.0), each adjusted to the target pH with phosphoric acid (85%, Fisher). This buffer was filtered prior to use through a 0.22μM filter membrane under vacuum and degassed through an ESA model DG4 degasser, and pumped at a rate of 0.5 ml/min, producing a background pressure of approximately 161 BARs.

Empirical data demonstrated good separation of ATP, ADP, AMP and ADO upon elution only with the Thermo-Fisher (previously ESA), DHBA-3.2×250 mm, C18, 5 micron particle size column in combination with the newly formulated mobile phase (Pani mobile phase, pH 2.75).

### Quantitative Analysis of Adenosine, ATP, ADP and AMP

**Figure 1:**
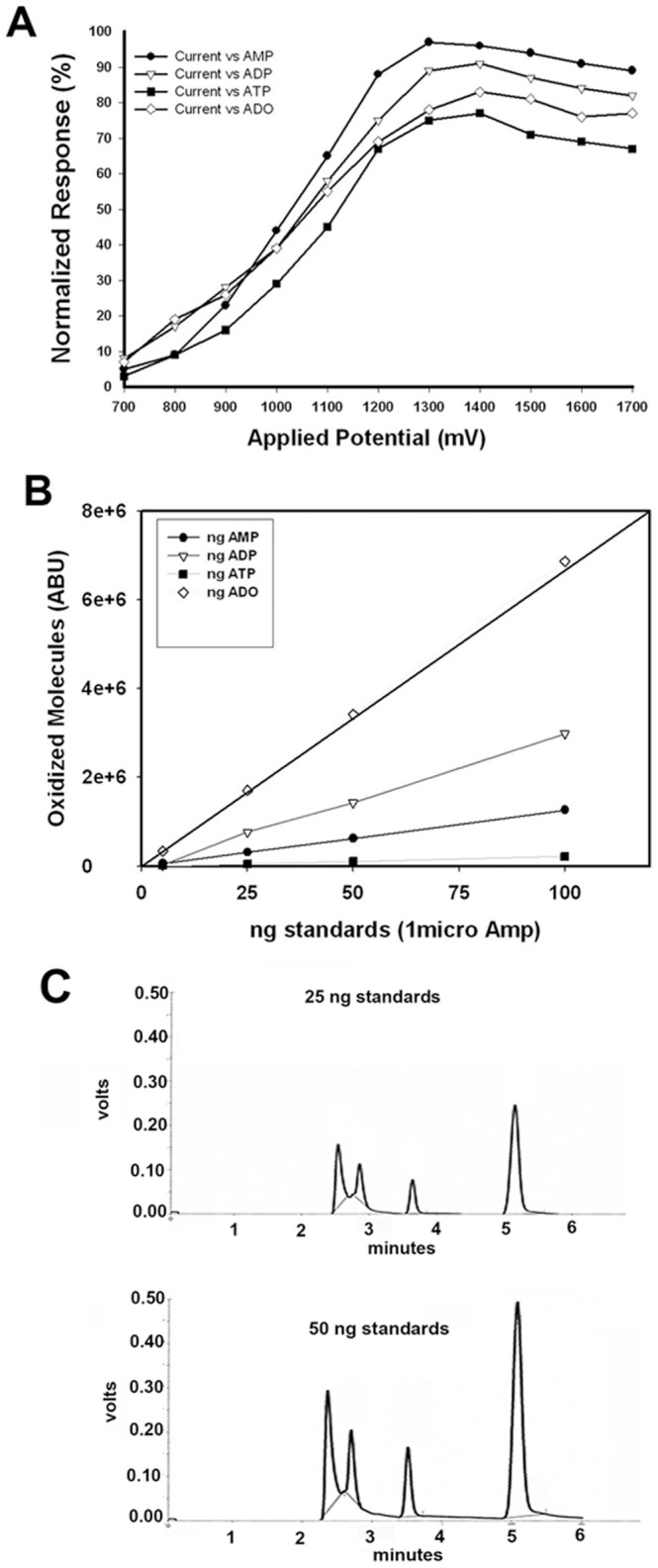
Identification of optimal parameters for simultaneous detection of ADO, ATP, ADP and AMP. (A) To determine the optimum voltage necessary to fully oxidize ADO, ATP, ADP and AMP, we measured the number of oxidized molecules at voltages ranging from +700–+1700 mV. Construction of a hydrodynamic voltamogram (HDV), necessary to determine the optimal signal/noise (s/n) ratio, showed that +1300 mV was optimum for the simultaneous detection of the compounds. (B) To determine the accurate range and linearity of detection of ADO, ATP, ADP, and AMP, 0–100 ng of each standard was injected into the HPLC. Electrochemical detection of the oxidized molecules shows a linear slope for each of the compounds. (C) Representative chromatograms of simultaneous separation of ADO, ATP, ADP and AMP demonstrating linearity of the injected analytes.

Adenosine, ATP, ADP and AMP were simultaneously detected and analyzed using HPLC-ECD. Briefly, the chromatographic system consisted of a solvent delivery system (Thermo-Fisher Scientific, (previously ESA), model 584 pump) and an autosampler (Thermo-Fisher Scientific, (previously ESA), model 542) equipped with an injection valve containing a 15 μl sample loop. ADO, ATP, ADP and AMP detection was accomplished by means of a coulometric electrochemical detector (Coulochem III, Thermo-Fisher Scientific, (formally ESA)). The analytical cell (Boron Doped Diamond (BDD, Thermo Electron North America, LLC, Model 5040) was used versus a hydrogen/palladium reference electrode and was set to +1300 mV. This voltage was chosen by empirical measurement of all four compounds (Figure 1A) and then balancing the ratio of oxidative potential to noise at each voltage (Fig. S2). Chromatographic separations were performed on a DHBA column (diameter 250×3.2 mm, Thermo-Fisher Scientific) inline with a pre-column column (Thermo-Fisher), and the entire system was run at ambient temperature.

The signal from the electrochemical detector was recorded using a model SS420x integration device (Scientific Software Inc.) and the retention time of ADO, ATP, ADP and AMP standards were empirically determined. After identification of retention time for each compound, concentration curves, ranging from 0–100 ng, were generated (Fig. 1B) and linearity of detection of the analytes was confirmed (Fig. 1C). Identification of ADO, ATP, ADP and AMP were further confirmed by spiking random samples with external standards and finding no peak shift (Fig. S3). ADO, ATP, ADP and AMP concentrations in tissues were quantified by comparing the peak areas of the sample chromatograms with the external standard chromatograms. Additionally, we performed a re-extraction procedure by further homogenizing the tissue from the pellets generated during the initial extraction in order to determine if we had fully extracted ADO, ATP, ADP and AMP. HPLC-ECD analysis of the re-extracted supernatant found no ADO, ATP, ADP or AMP, and on this basis, we concluded that the peaks co-eluting from mice brain samples, in comparison to the external standards chromatogram, represent a true picture of the levels of ADO and its metabolites in the mouse brain. Differences in levels of ADO between strains were compared using One Way ANOVA with post hoc Bonnferoni’s multiple comparisons (Prism Version 5.0, Graphpad Software, LaJolla, CA).

## Results

### Determination of ADO, ATP, ADP and AMP

**Figure 2:**
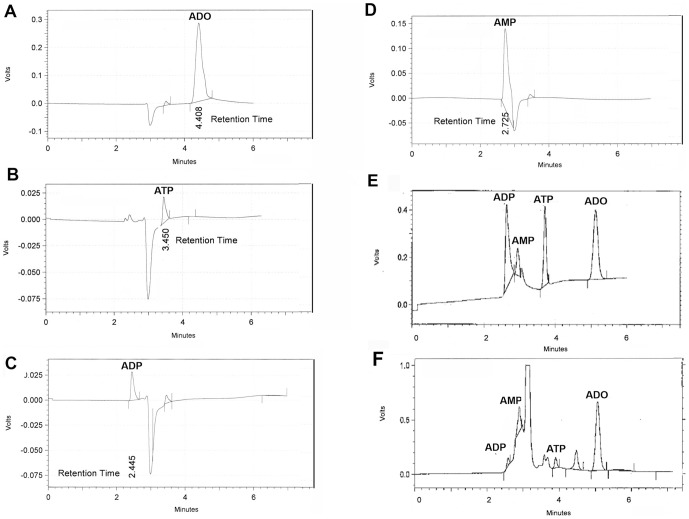
Determination of elution time for Purines. Sample chromatograms showing elution time for (A) ADO, (B) ATP, (C) ADP, (D) AMP, (E) a combined solution containing all four compounds and (F) Olfactory bulb from a 24 month C57BL/6J mouse. The elution time for each of the compounds is distinct and allows for the simultaneous detection of ADO, ATP, ADP and AMP.

To determine the retention time of ADO, ATP, ADP and AMP, purified compounds (adenosine 99% pure, Sigma, St. Louis; Adenosine triphosphate disodium (ATP, LKT laboratories, Cat # A1319, St. Paul, MN), Adenosine 5′-diphosphate (ADP, Sigma), and Adenosine 5′-monophosphate, (AMP, Sigma) were dissolved in the Pani mobile phase at 4 different pH values (1.75, 2.75, 3.75 and 7.0). Ten microliters of combined standards and/or samples at each pH were injected into the HPLC at +1300 mV with a flow rate of 0.5 ml/min. We found that the best separation and resolution of the peak amplitude occurred at pH 2.75 (Fig. S4), thus the Pani mobile phase used for all subsequent analysis was done at this pH. The retention times of ADO, ATP, ADP and AMP were then empirically determined (Fig. 2A-D). To demonstrate that each of these could be simultaneously detected on a single chromatogram, 10 microliters of a 1 mg/ml solution of an equal mixture of ADO, ADP, AMP and 10 mg/ml ATP was analyzed. Four distinct peaks showing the individual compounds were resolved and there was no significant difference in the HPLC-ECD retention times when compared to their individual elutions (Figure 2E) or biological samples (Figure 2F).

### Quantification of ADO, ATP, ADP and AMP in C57BL/6J brain

The levels of ADO, ATP, ADP and AMP (pg/mg wet weight of tissue) in 6 different brain regions of C57BL/6J mice and their contribution to the total pool of purines are shown in Table 1. Of the six regions examined, the striatum and hippocampus have the highest amounts of total ADO, with cerebral cortex and substantia nigra having intermediate levels, while the olfactory bulb and cerebellum has the lowest levels measured. A similar pattern was seen in ATP, ADP and AMP pools.

**Table 1:** Total Adenosine, ATP, ADP, and AMP (pg/mg wet weight) in Brain Regions of 12 Month C7BL/6J mouse.

Brain Region	Adenosine	ATP	ADP	AMP	Total Pool
Olfactory Bulb	3041±473	29865±1941	16684±2058	618096±48753	667686
% of total	1	5	2	96	
Cerebral Cortex	12620±2065	89799 ±14699	12620±4462	56682±4274	171721
% of total	7	52	7	34	
Striatum	184669±25677	682872±65889	289646 ±15151	1582489±75113	2739676
% of total	7	24	11	58	
Hippocampus	150597±29364	1673536±194710	938762±1392562	938762±1392562	3232798
% of total	5		29	29	
Substantia nigra	16029±2265	92867±4860	308429±23553	308429±23553	3232798
% of total	3	19	65	65	
Cerebellum	9111±1877	35650± 5205	26887±3009	26887±3009	99166
% of total	9	36	27	27	

### Quantification of ADO in the CNS of different mouse strains

**Figure 3:**
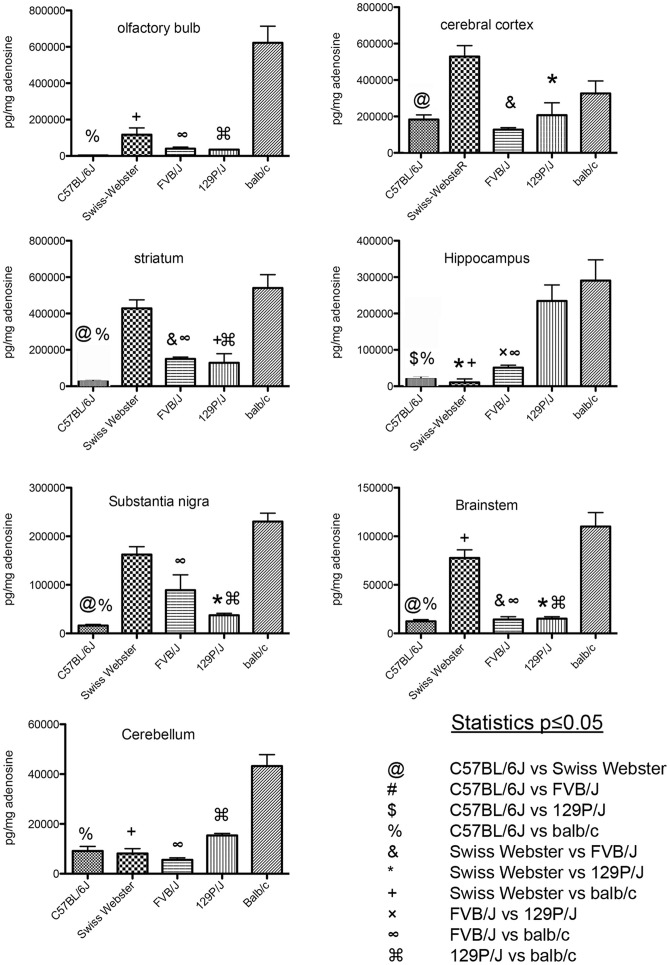
Quantitation of ADO in 7 regions of the brain in 5 strains of mice. ANOVA demonstrated significant differences between mouse strains in each region examined. Post-hoc Bonferroni comparisons are shown for each brain region. N = 10 for each measurement.

The levels of ADO (pg/mg wet weight of tissue) in 7 different brain regions of five different strains mice are shown in Figure 3. The statistical significance of the changes among the strains and regions was assessed by ANOVA followed by Bonferroni post hoc analysis using SigmaStat software.

In general, the levels of ADO in the brain of each of the strains examined at basal conditions were low, with the exception of the BALB/c strain that had relatively higher ADO levels. In addition to differing levels of ADO among strains, there was also a variation dependent on the region examined. Regionally, highest levels of ADO were seen in the striatum and olfactory bulb and lowest levels in the brainstem. We found significant differences among mouse strains in each region examined (Figure 3A-G).

In olfactory bulb (Figure 3A), significant differences were seen between the 5 strains (F = 32.57, p≤0.0001) with ADO in BALB/c significantly increased compared to the other 4 strains examined. The order of ADO from highest to lowest in the olfactory bulb was BALB/c→SW→FVB/NJ→129P/J→C57BL/6J˜. The increased ADO in BALB/c ranged from 532% higher than that measured in SW to a 17,566% increase compared to C57BL6/J.

In the striatum (Figure 3B), significant differences were noted between the 5 mouse strains (F = 26.67, p≤0.0001), with the highest levels of ADO in SW and BALB/c and lowest levels in C57BL6/J. The order of ADO from highest to lowest in striatum was BALB/c→SW→FVB/NJ→129P/J→C57BL/6J˜. The increased ADO in BALB/c ranged from 162% higher than that measured in BALB/c to a 2480% increase compared to C57BL/6J.

In the cerebral cortex (Figure 3C), significant differences were noted between the 5 mouse strains (F = 9.322, p≤0.0001) with the higher levels of ADO in SW and BALB/c and lower levels in 129P/J, C57Bl/6J FVB/NJ. The order of ADO from highest to lowest in the cerebral cortex was SW→BALB/c→129P/J→C57BL/6J→FVB/NJ˜. The increased ADO in SW ranged from 255% higher than that measured in 129P/J to a 415% increase compared to FVB/NJ.

In hippocampus (Figure 3D), significant differences were noted between the 5 mouse strains (F = 16.70, p≤0.0001), with the highest levels of ADO in 129P/J and BALB/c and lowest levels in SW. The order of ADO from highest to lowest in hippocampus was BALB/c→129P/J→C57BL/6J→FVB/NJ→SW˜. The increased ADO in BALB/c ranged from 567% higher than that measured in 129P/J to 2787% increase compared to SW

In substantia nigra (Figure 3E), significant differences were noted between the 5 mouse strains (F = 22.73, p≤0.0001), with the highest levels of ADO in SW and BALB/c and lowest levels in C57BL/6/J and 129P/J. The order of ADO from highest to lowest in substantia nigra was BALB/c→SW→FVB/NJ→129P/J→C57BL/6J˜. The increased ADO in BALB/c ranged from 258% higher than that measured in FVB/NJ to a 701% increase compared to C57BL/6J.

In brainstem (Figure 3F), significant differences were seen among the 5 groups (F = 39.81, p≤0.0001), with the highest levels of ADO in SW and BALB/c and lowest levels in C57BL/6/J. The order of ADO from highest to lowest in brainstem was BALB/c→SW→FVB/NJ→129P/J→C57BL/6J. The increased ADO in BALB/c ranged from 951% higher than that measured in FVB/NJ to an 1139% increase compared to C57BL/6J.

In cerebellum (Figure 3G), significant differences were seen among the 5 groups (F = 44.20, p≤0.0001), with the highest levels of ADO in BALB/c and lowest levels in C57BL/6/J. The order of ADO from highest to lowest in brainstem was BALB/c→SW→C57BL/6J→SW→FVB/NJ. The increased ADO in BALB/c ranged from 281% higher than that measured in 129P/J to a 793% increase compared to FVB/NJ.

## Discussion

Although adenosine has been implicated in a number of important biological processes, its direct detection and quantitation in tissue has been difficult. For this reason, much of the research examining the biological function of adenosine has relied on downstream effects of signaling, through its known receptors. While this approach has clearly demonstrated the importance of adenosine in many aspects of neurological function, little is known regarding its regional expression and concentrations in the brain, or if there are differences in ADO levels among mouse strains. The former is important since behaviors, and even diseases, are manifested by a loss/gain of function in specific brain structures, while the latter is important since it has been shown that background strain is a critical variable when designing and engineering mouse models of human disease.

There have been a few reports of direct measurement of adenosine using HPLC with UV detection [32], [33], most of the methods described for measuring ADO and its metabolites rely on indirect measurements. Dale et al [34] developed a sensor that measured adenosine indirectly based on successive breakdown of adenosine to inosine to xanthine, which was then oxidized to uric acid that produced hydrogen peroxide. Adenosine levels were then indirectly estimated by the electrochemical detection of this final product. Howard et al [35] used HPLC and fluorimetry to indirectly estimate adenosine levels by measurement of a fluorescent analogue of adenosine, 1,N^6^-ethenoadenosine triphosphate [36]. Kloor et al [37], developed a simple and sensitive binding assay to indirectly detect adenosine based on the displacement of ^[3H]^adenosine from the reduced form of *S*-adenosylhomocysteine hydrolase.

Although total ADO (both intracellular and extracellular) in tissue has been difficult to measure, several studies have successfully examined ADO release and extracellular concentrations via fast scan cyclic voltammetry (FSCV) [38], [39].

Recently, Birbeck and Mathews, [40] as in this paper, reported the use of a BDD electrode to simultaneously detect dopamine and adenosine from tissue. Like our results, they found that standard mobile phases were less than optimal to detect adenosine (although they did detect dopamine). They developed a mobile phase consisting of acetonitrile, ammonium phosphate, and sodium pyrophosphate; after which they could simultaneously detect all of the catacholamines and their metabolites as well as AMP, but not ATP and ADP. The simultaneous detection of both catacholamines and ADO is useful in that one can use a single tissue sample, prepared the same way to extract information regarding multiple neurochemical profiles, but since the oxidation potentials of dopamine and ADO are very different, one clearly has to compromise on detection levels (see Birbeck and Mathews, Figure 2, where the chosen voltage is 25% of the ADO). Thus, using this method, Birbeck and Mathews reported a limit of detection of 1.2nM tissue ADO and 0.021 nM dopamine, while using our method-optimized for purines-we can detection 37x higher ADO (approximately 44 pM). Additionally, this optimized method allows one to also simultaneously detect the ADO metabolites, ATP, ADP and AMP.

Adenosine has been shown to have a number of important functions. Although not typically thought of as a classical neurotransmitter (since it does not appear to be synthesized or stored in vesicles), it does appear to have a modulatory role on the release or other transmitters and can have profound effects far from its point of origin [41]. Modulation of adenosine and its signaling have been implicated in a number of neurological disorders including epilepsy, stroke, schizophrenia, Alzheimer’s and Parkinson’s disease [5], [8], [42], [43]. Adenosine signals through a number of different receptors, classified as P1 and P2 [44]. However, any function ascribed to adenosine will necessarily have its effect through the combinatorial activation of any number of these receptor subtypes.

Our findings show that there is a tremendous variability in the total ADO levels among different strains of mice. Since our measurements are taken from whole brain, the measured ADO is cumulative from both intracellular and extracellular stores. Additionally, the measured ADO levels are a combination of the additive actions of adenosine formation and breakdown; and thus are only a snapshot of total adenosine content. The formation of adenosine is controlled by the enzymatic activity of intracellular and extracellular AMP-selective 5′ nucleotidases that catalyze the breakdown of AMP combined with adenosine release from S-adenosylhomocysteine via the actions of S-adenosylhomocysteine hydrolase [45]. Breakdown of adenosine is modulated by the combined actions of adenosine kinase that modulates conversion of AMP by addition of a phosphate group to adenosine and adenosine deaminase that dephosphorylates adenosine to inosine [46]. Due to the interplay of these enzymatic systems, the purine pool is labile and changes occur rapidly, often times changing their state in less than 1 second [47]. Thus, to reiterate, one must be aware that the chromatographic measure of adenosine occurs as a snapshot of one time; but we feel that the relative levels are comparable across strains given that the period between sacrifice and stopping enzymatic activity by freezing was equal and controlled among each of the strains examined. A better measurement of actual pool values might occur with other methods of tissue processing, such as the use of microwave fixation [48]; however, this procedure was not available at our facility.

As observed with ADO pools, the levels of ATP, ADP and AMP also differed depending on the regions of the C57BL/6J brain examined. To determine that these differences were not due to alterations in form, we also examined the total purine pool (pg/mg ADO + ATP + ADP+ AMP), and found that this total pool is dependent on brain region and appear to be unrelated to total ADO levels. For example, we see that in regions of high ADO (striatum with 184669 pg/mg) the ADO comprises 7% of the total nucleotide pool, while in the cerebellum (9111 pg/mg, 5% of striatal ADO), the ADO:nucleotide pool ratio is 9%. The lowest ADO:nucleotide pool ratios were seen in the olfactory bulb and substantia nigra (0.45% and 0.33%, respectively), which each had the highest ratio of AMP: total nucleotide pool (96% and 65%, respectively). It is unclear at this time, what the significance of the ATP, ADP, AMP and ADO pool percentages are in relation to ADO function, although one might conceive that the areas with highest ATP pool are more metabolically active (cerebral cortex, hippocampus and cerebellum), while areas with higher AMP pools (olfactory bulb, striatum, substantia nigra), which could directly convert to ADO and contribute to activation of P1R and P2R receptors and modulation of other neurotransmitters [49], [50], are in a state of plastic readiness (especially since these experiments were done during a physically inactive period (daytime). Additionally, AMP and ADP have been shown to be a direct allosteric activator of AMP-activated protein kinase (AMP), which acts as a direct energy sensor in the eukaryotic cells [51].

Functionally, ADO acts as a neuromodulator, and thus the differences in the purine levels we measured could affect a wide variety of neurotransmitter and neurohormone systems that underlie strain related differences in behavior. Historically there have been only few studies that examined regional differences in ADO levels in brain. Kovacs et al [52] using a chromatography based assay found high levels of ADO in cerebral cortex with low levels in thalamus; and even within these regions there was variability. Kobayashi [53] examined ADO in decapitated guinea pig brain and found high levels in hippocampus, thalamus and cortex, intermediate values in tectum and low levels in cerebellum. Gharib [32] found high levels of ADO in cortex and striatum with lower levels in mesencephalon and brainstem and cerebellum. Caballos postulated that these changes in regional ADO levels might relate to the ratio of neuron to glia in each region [54].

In this study we observed ADO levels in a number of brain regions and compared them across different strains of mice. In the olfactory bulb, ADO levels were fairly low, except in the BALB/c (600 ng/mg) strain. Expression studies of adenosine receptors demonstrate moderate but specific A2aR expression in the granule cell layer [55] and the mitral cell layer [56], while A1aR in the olfactory bulb is expressed primarily in the mitral cell layer [56]. Olfaction in mice plays many functional roles including providing cues for navigating through their environment, interpreting social interactions, as well as providing cues that contributing to learning and memory. In regard to strain differences in olfaction, Restivo et al found that BALB/c mice were superior to other strains of mice (including the C57BL/6J strain) in olfactory-based learning [57]. Additionally, Lee et al found that BALB/c mice had an order of magnitude greater odor sensitivity than 129/S1 and C57BL/6J mice [58].

Similar to the measurement of ADO in the olfactory bulb, we observed a dichotomy of ADO levels in the cerebellum; such that BALB/c had significantly higher levels of ADO than any other strain examined. Expression maps from the Allen Brain Atlas show very low levels of expression of the A1aR or A2aR receptors in the cerebellum [56], although A1R has been reported in cerebellar granule cells [59] with particular expression in parallel fibers [60] and on the surface of Purkinje and deep nuclear cells [61]. The role of adenosine signaling in the cerebellum has not been well studied. Functionally, the cerebellum plays a major role in coordination of fine motor control as well as in aspects of motor learning; and these processes are mediated, in part, through NMDA receptors [62]–[64]. Adenosine and NMDA receptors are functionally related, whereby activation of glutamatergic NMDA channels suppresses the inhibitory actions of adenosine [65]. In terms of strain differences in cerebellar response, Bao et al showed that conditioned eye blink response, a behavior associated with cerebellar learning was far superior in BALB/c mice than C57BL/6J mice [66] and is related to function of NMDA channels [64], [67].

Like other regions in the brain, ADO levels vary by strain in the basal ganglia, with Swiss-Webster and BALB/c strains having significantly higher levels than C57BL/6J, FVB/NJ and 129P/J. Like glutamate, adenosine and dopamine are also coregulated; and the coordination of these neurochemicals is thought to underlie both normal and pathological functions. Mechanistically, it appears to be that stimulation of A1R and A2R receptors by ADO [68] or ADO analogues [69], [70] reciprocally alters the binding characteristics of D_1_ and D_2_ receptor signaling [71]–[74]. D1 receptors are generally thought to be predominant in the “direct” pathway (caudate to GPi/SNr to thalamus to motor cortex; activation causing release of GABA and inhibition of firing) while D2 receptors are predominant in the “indirect” pathway (caudate to GPe to subthalamic nucleus to GPi/SNr to thalamus to motor cortex; activation causing release of GABA and inhibition of firing) [75]. Studies have shown that activation of A2A receptors reduced the binding and signaling of D2 receptors while activation of A1A receptors inversely modulated D1 binding and activation [76], [77]. Thus, lower levels of ADO would lead to higher levels of dopamine signaling, increased GABA release and potentially lowered levels of basal ganglia activity, while higher levels of ADO would lead to activation of these pathways resulting in higher dopamine release.

Decreased activity in the basal ganglia is thought to underlie some of the primary symptoms (tremor, bradykinesia) that are observed in Parkinson’s disease. Experimentally, a number of studies have shown that adenosine receptor antagonists can reduce tremor, improve motor output and may even be neuroprotective [78]–[80], and for this reason these receptors have been targets for the treatment of parkinsonism. It is interesting that in this study, the mouse strains that had lower levels of ADO are those that are generally more sensitive to xenobiotic agents used in modeling of Parkinson’s disease, including MPTP and paraquat [81]–[84]. Given that recent studies have shown that dopamine can act as an inflammatory molecule in the brain [85] and that excess levels of dopamine can readily autoxidize to form free radicals [86], it is possible that differing levels of ADO pools may play a role in how dopamine is modulated an thus those individuals or animals with higher pools of basal ganglia ADO may be less prone to developing Parkinson’s disease [87], [88].

In summary, we describe an HPLC-ECD based method for the direct, simultaneous and accurate detection of ADO, ATP, ADP and AMP isolated from fresh mouse brain tissue. We find that there are significant differences in ADO levels in different subregions of the brains between strains of mice; and these differences may underlie the various behavioral and neurodegenerative responses when used in experimental disease models.

## Supporting Information

Figure S1 **Percent recovery of purines in standard diluents. Known amounts of ADO, ATP, ADP and AMP were mixed in Pani mobile phase (MP), perchloric acid (PCA) or a combination of 0.3N PCA with Pani mobile phase (25%PCA:75% mobile phase).** Recovery was approximately 90% in PCA/mobile phase that was then used for each sample.
(TIF)

Figure S2 **Comparison of oxidative potential to noise at different voltages.** Chromatograms of 10 ul of injected standards with a flow rate of 0/5 ml/min (25 ng each of ADP at 2.625 min, AMP at 2.981 min, ATP at 3.703 min and ADO at 5.108 min; each at 25 ng/ml) run at 1300mV or 1400 mV. At 1300 mV there is a slightly better peak resolution and less noise in the samples (not shown) compared to 1400 mV.
(TIF)

Figure S3 **Coelution of sample peaks with added standards.** (A) Chromatogram of purine detection from 24 month C57BL/6J olfactory bulb. Arrows mark ATP and ADO peaks. Unknown peaks are marked by *. (B) The sample from (A) was spiked with 25 ng each of ADO, ATP, ADP and AMP. The resultant chromatogram shows a clear increase in ATP and ADO (arrows), while the peaks of the unknown compounds (*) are unaffected. AMP and ADP in the spiked sample are lost in the peak of the solvent front.
(TIF)

Figure S4 **Effect of pH on peak resolution.** Chromatograms of 10 ul of 25 ng/ml injected standards with a flow rate of 0/5 ml/min at four different pH values. At pH 2.75 the separation and peak resolution is optimal.
(TIF)
