# Supplementary figures and images for: Neurochemical Measurement of Adenosine in Discrete Brain Regions of Five Strains of Inbred Mice
Source: PLoS One. 2014 Mar 18;9(3):e92422. doi: 10.1371/journal.pone.0092422 (PMC3958516; doi:10.1371/journal.pone.0092422)

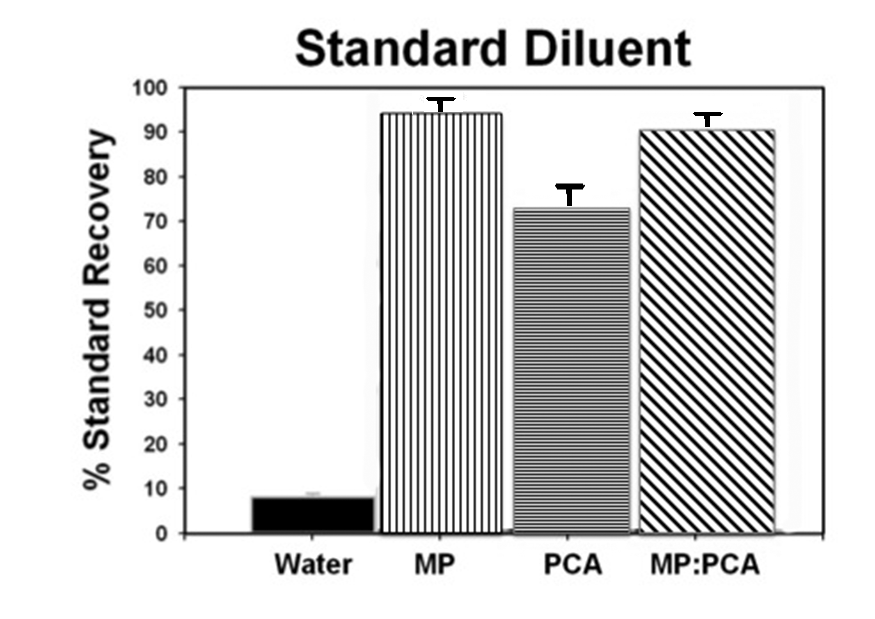

Supplement: Figure S1 — Percent recovery of purines in standard diluents. Known amounts of ADO, ATP, ADP and AMP were mixed in Pani mobile phase (MP), perchloric acid (PCA) or a combination of 0.3N PCA with Pani mobile phase (25%PCA:75% mobile phase). Recovery was approximately 90% in PCA/mobile phase that was then used for each sample. (TIF) [file pone.0092422.s001.tif]

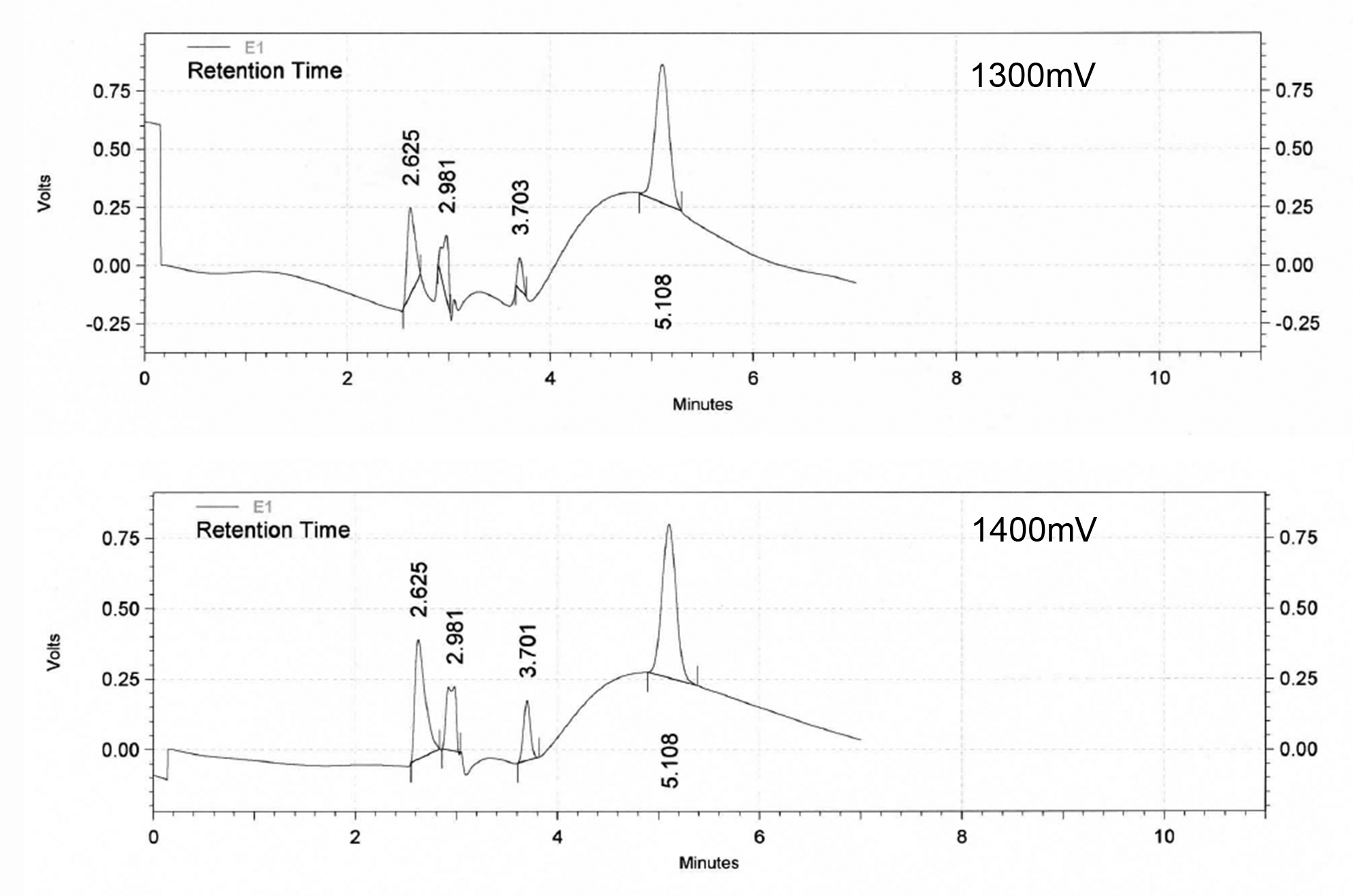

Supplement: Figure S2 — Comparison of oxidative potential to noise at different voltages. Chromatograms of 10 ul of injected standards with a flow rate of 0/5 ml/min (25 ng each of ADP at 2.625 min, AMP at 2.981 min, ATP at 3.703 min and ADO at 5.108 min; each at 25 ng/ml) run at 1300mV or 1400 mV. At 1300 mV there is a slightly better peak resolution and less noise in the samples (not shown) compared to 1400 mV. (TIF) [file pone.0092422.s002.tif]

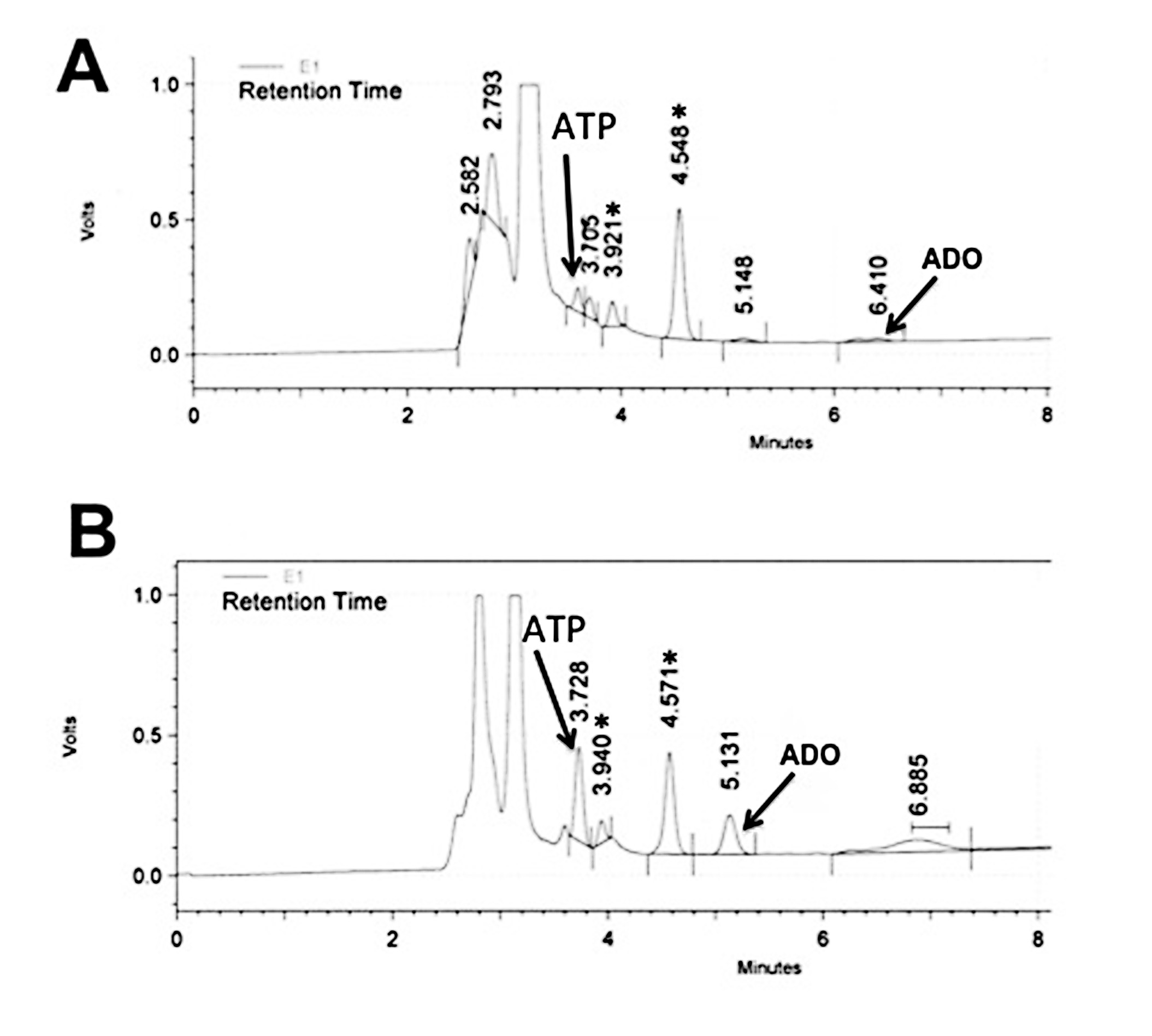

Supplement: Figure S3 — Coelution of sample peaks with added standards. (A) Chromatogram of purine detection from 24 month C57BL/6J olfactory bulb. Arrows mark ATP and ADO peaks. Unknown peaks are marked by *. (B) The sample from (A) was spiked with 25 ng each of ADO, ATP, ADP and AMP. The resultant chromatogram shows a clear increase in ATP and ADO (arrows), while the peaks of the unknown compounds (*) are unaffected. AMP and ADP in the spiked sample are lost in the peak of the solvent front. (TIF) [file pone.0092422.s003.tif]
